# Enantioselective Self-Assembled Nanofibrillar Network with Glutamide-Based Organogelator

**DOI:** 10.3390/nano11061376

**Published:** 2021-05-23

**Authors:** Nao Nagatomo, Hisashi Oishi, Yutaka Kuwahara, Makoto Takafuji, Reiko Oda, Taisuke Hamada, Hirotaka Ihara

**Affiliations:** 1 Department of Applied Chemistry and Biochemistry, Kumamoto University, 2-39-1 Kurokami, Chuo-ku, Kumamoto 860-8555, Japan; kumacats01@gmail.com (N.N.); kumacats02@gmail.com (H.O.); kuwahara@kumamoto-u.ac.jp (Y.K.); 2 Institut de Chimie & Biologie des Membranes & des Nano-objects, CNRS, 33607 Pessac, France; reiko.oda@u-bordeaux.fr; 3 National Institute of Technology, Okinawa College, 905 Henoko, Nago, Okinawa 905-2192, Japan; hamada@okinawa-ct.ac.jp

**Keywords:** molecular gel, nanofibril, self-assembly, secondary chirality, enantioselectivity, circular dichroism

## Abstract

A chiral molecular gelation system, as a chiral host, was used to effectively realize enantioselectivity using the simple carboxylic acid functional group. For this purpose, an L-glutamic-acid-based lipidic amphiphile (*G*-CA) with a carboxylic head group was selected and its responsiveness to cationic guest molecules was investigated. The dispersion morphology of *G*-CA in its solution state was examined by confocal and transmission electron microscopies, while interactions between the *G*-CA, as the host system, and guest molecules were evaluated by UV-visible, circular dichroism, and fluorescence spectroscopies. As a result, enantioselectivity was effectively induced when *G*-CA formed highly ordered aggregates that provide negatively charged surfaces in which carboxyl groups are assembled in highly ordered states, and when the two cationic groups of the guest molecule are attached to this surface through multiple interactions.

## 1. Introduction

Chiral molecules are the basis of life [1]. Primary chirality is amplified to higher chirality by the precise orientations and arrangements of molecular chiral units, which produces a variety of life-based phenomena. In synthetic chemistry, chiral synthesis and chiral separation are still evolving as universal research tools, with supramolecular organogels attracting attention as media for chiral separation in recent years. Such gelation, unlike partially crosslinked polymer gelation, is characterized by the formation of a three-dimensional network through one-dimensional growth involving molecular aggregates of small chiral molecules, such as amino acids [2,3,4,5] and other chiral organic sources [6,7,8,9]. Therefore, such gels are very attractive in that they form highly ordered chiral orientations that are difficult to obtain using common polymer gels.

**Figure 1:**
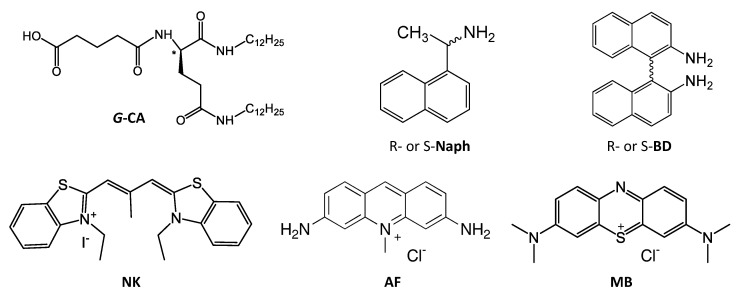
Chemical structures of key materials used in this work. L-glutamic-acid-based organogelator (*G*-CA): L-glutamic-acid-based organogelator; NK, AF, and MB: cyanine dye NK-77, acryflavine, and methylene blue as cationic fluorescent dyes, respectively; Naph and BD: 1-naphthylethylamine and binaphthyl-2,2’-diamine, respectively.

The first examples of chiral separation using organogels were reported as enantioselective transport phenomena of amino acid derivatives from gels to aqueous phases [10]. Since then, most of the remarkable enantioselectivities obtained using chiral organogels have been realized by the introduction of special supramolecular or multi-functional groups, such as a porphyrin [11,12], a pyridylpyrazole [13], and a quinolinol [14] into a molecule. In this study, we aimed to induce enantioselectivity through amplification of chirality using simple functional groups in a manner that does not rely on a supramolecular functional group. Inducing enantioselectivity using a carboxyl group as the simplest functional group was the main challenge of this study (Figure 1).

## 2. Materials and Methods

### 2.1. Materials and Reagents

We synthesized the L-glutamic-acid-based organogelator (*G*-CA) according to the method reported previously by us [15]. Cyanine dye NK-77 (NK), acriflavine (AF), and methylene blue (MB), as fluorescent dyes, were purchased from Photosensitizing Dyes Co., Ltd., Tokyo Chemical Industry Co, Ltd., and Tokyo Chemical Industry Co, Ltd., respectively, and were used as received. Binaphthyl-2,2’-diamine (BD) and 1-naphthylethylamine (Naph), as chiral guest molecules, were purchased from Sigma-Aldrich, Inc. and Tokyo Chemical Industry Co, Ltd., respectively, and were used as received.

### 2.2. Preparing the G-CA assembly

*G*-CA was dissolved in hot toluene or methylcyclohexane (80 °C). The solution was cooled and left at 25 °C for 30 min to obtain a clear solution or gel. The dye solution was prepared at high concentration in THF, after which a small portion was dissolved in the *G*-CA solution. After mixing the *G*-CA solution with the dye solution in the required ratio, triethylamine was added to the mixture in quantities of up to 10 molar equivalents relative to *G*-CA.

### 2.3. Characterization

UV–visible absorption, circular dichroism (CD), and fluorescence (FL) spectra were acquired using V-560 (JASCO, Tokyo, Japan), FP-6500 (JASCO, Tokyo, Japan), and J-725 instruments (JASCO, Tokyo, Japan), respectively. Confocal microscopy and transmission electron microscopy (TEM) images were acquired on TCS 8SP (Leica Microsystems, Wetzlar, Germany) and JEM-1400 Plus microscopes (JEOL, Tokyo, Japan), respectively. The sample for TEM observation was prepared as follows: The *G*-CA-containing cyclohexane solution was dropped onto a polymer-coated copper mesh, the excess liquid was removed with a filter paper, and then air-dried. An aqueous solution of uranyl acetate was dropped onto the obtained copper mesh, excess liquid was removed similarly, and finally dried *in vacuo*.

## 3. Results and Discussion

### 3.1. Detecting Nano-fibrillar G-CA Aggregation 

In this study, *G*-CA (shown in Figure 1) was selected as the low-molecular organogel-forming compound. *G*-CA is a carboxyl-group-containing derivative of L-glutamic acid that promotes the formation of chiral assembly. In addition to its simple structure, the advantageous properties of *G*-CA can be emphasized by good solubility in various organic solvents and compatibility for hydrophobic polymer materials.

**Figure 2:**
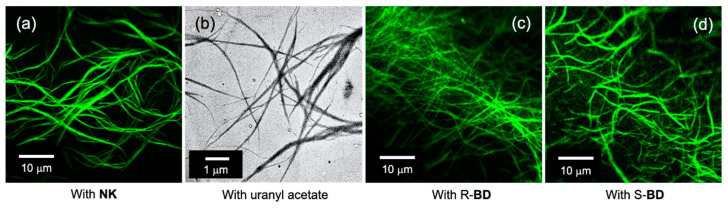
(**a**,**c**,**d**) *G*-CA aggregation in solution at 25 °C observed by confocal laser microscopy and (**b**) a TEM image of a dried sample of the *G*-CA solution. The confocal images were acquired in the presence of (**a**) NK and the (**c**) R- and (**d**) S-enantiomers of BD. The dried sample of *G*-CA was prepared by casting and drying on a copper mesh plate. Concentrations: (**a**) *G*-CA, 0.5 mM; dye, 0.025 mM, (**b**) *G*-CA, 0.75 mM; dye, 0.05 mM. Solvents: (**a**) toluene and (**c**,**d**) 95:5 (v/v) methylcyclohexane:THF.

Toluene and methylcyclohexane, which were observed to lead to aggregation at low concentrations, were selected as the main solvents. Figure 2a shows a confocal laser micrograph of a 0.5 mM toluene solution of *G*-CA. Since *G*-CA is not fluorescent, NK, a cationic cyanine dye, was added as a luminescent component for imaging purposes. The observed fibrous luminescence suggests that *G*-CA had aggregated and that NK was electrostatically bound to it.

The *G*-CA dispersion appeared as an apparent solution in the 0.5–0.75 mM concentration range; however, it gelated at a concentration of 1 mM and above. These observations indicate that *G*-CA forms nanofibrillar aggregates through self-assembling-induced one-dimensional growth and the fibrous aggregates intertwine to form the three-dimensional network required for gelation with increasing concentration.

The actual diameters of the fibrous aggregates were estimated by subjecting the dried sample of the *G*-CA solution to TEM. As shown in Figure 2b, thin fibers with minimum diameters of several tens of nanometer were observed. Since it is considered that drying can promote bundling with nanofibrillar aggregates, we conclude that the observed fibrous aggregates are dispersed as several tens or less of nanometer in diameter in the *G*-CA solution.

### 3.2. Characterizing G-CA as a Chiral Host System

Glutamic-acid-derived aggregates are well known to interact with guest molecules to induce chiral perturbations [16]. In this study, the chiral functionality of the *G*-CA aggregate was investigated using the three non-chiral dyes shown in Figure 1.

**Figure 3:**
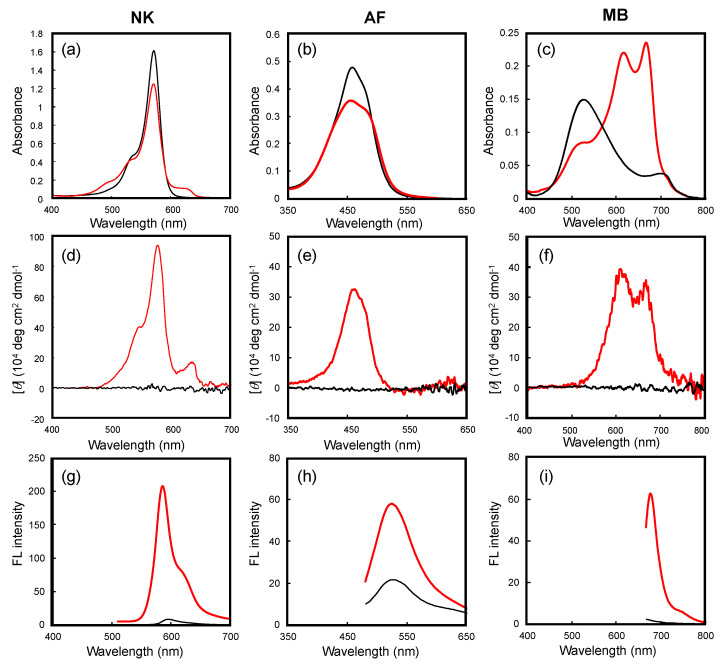
(**a–c**) UV-visible, (**d–f**) CD, and (**g–i**) FL spectra of dyes in the presence and absence of *G*-CA aggregates at 25 °C. Solvent, toluene; concentrations: 0.05 mM (dye), 0.75 mM (*G*-CA), 0.5 mM (TEA). The red and black traces correspond to the presence and absence of *G*-CA, respectively. The FL spectra in (**g–i**) were obtained by excitation at 500, 470, and 650 nm, respectively.

Figure 3 shows typical spectra of the three dyes in toluene in the presence and absence of *G*-CA. CD was not observed in the absence of *G*-CA, as shown by the black traces in Figure 3d–f because the dyes themselves are not optically active. On the other hand, distinct CD signals were observed around the absorption bands of the dyes when *G*-CA was added with triethylamine (TEA). Since no significant CD was induced in the absence of TEA, we presume that TEA neutralizes the counter anion of the cationic dye to promote the electrostatic interaction between the weakly acidic carboxyl groups of *G*-CA and the cationic dyes.

When the solvent was replaced from a cyclohexane system to chloroform, similar CD induction was not observed, irrespective of whether TEA was added or not. This is related to the fact that chloroform is a good solvent of *G*-CA, and therefore the highly ordered state cannot be produced in the same condition. In addition, significantly weaker CD signals were observed in methylcyclohexane when a few equimolar drops of trifluoroacetic acid, which inhibits hydrogen bonding interactions in *G*-CA, were added. These observations led us to conclude that the dye/*G*-CA interactions that induce CD in the non-chiral dyes is a specific phenomenon that occurs only when *G*-CA is aggregated rather than in its monomeric dispersed state.

As shown in Figure 3g–i, dye fluorescence was significantly more intense in the presence of the *G*-CA aggregates. The aggregation-induced emission enhancement (AIEE) mechanism [17,18,19,20] has been proposed to explain such a phenomenon. However, as shown in Figure 3a–c, since the absorption spectral behaviors are significantly different between MB and the other dyes, it is difficult to explain the increase in fluorescence intensity only by AIEE: the absorption spectrum of MB exhibited a remarkable red shift with increasing absorption, while no significant changes were observed for the absorption maxima of NK and AF; however, they exhibited slight reductions in absorption intensity.

In order to explain these different observations, we discuss the differences in the microenvironments of the dyes as guest molecules on *G*-CA aggregates. The red shift observed for MB is reminiscent of the formation of dimeric stacking. In general, a head-to-head type-*H* association of a dye leads to quenching, but a head-to-tail type-*J* association amplifies fluorescence [21]; hence, we presume that *G*-CA aggregates promote the phase transition of MB from monomeric to a *J*-like association. The resulting CD signal (Figure 3f) is also observed near the absorption band corresponding to the newly generated *J*-associated species. On the other hand, almost no peak shift was observed for either NK or AF. Therefore, we conclude that these dyes do not dimerize on the *G*-CA aggregates and that fluorescence enhancement is most likely due to microenvironmental changes of the dye, which are affected by the polarity of the environment, with a polar environment promoting the integration of the carboxyl groups along the fibrillar aggregates.

### 3.3. Enantioselective Response of the G-CA Aggregates

The enantioselectivity of *G*-CA was evaluated using the enantiomers of 1,1’-binaphthyldamine (R- and S-BD). Figure 2c,d show confocal microscope images of *G*-CA in the presence of the R- and S-enantiomers of BDs, respectively. Distinct fluorescent fibrillar images were observed in both solutions. While no significant difference is observed between R- and S-BD, the fluorescence images clearly reveal interactions between *G*-CA and the fluorescent BD due to no fluorescence in *G*-CA alone.

**Figure 4:**
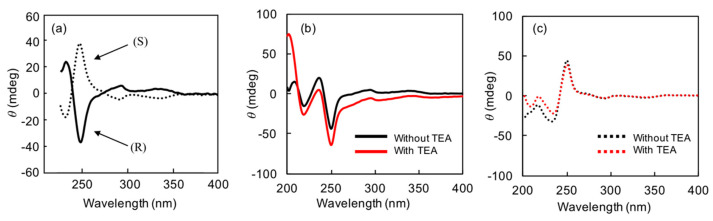
(**a**) CD spectra of BD alone. The effect of TEA on the enantioselective responses of (**b**) R-BD and (**c**) S-BD in the presence of *G*-CA. Solvent: 95:5 (v/v) methylcyclohexane:THF. Concentrations: *G*-CA, 0.75 mM; BD, 0.05 mM. Temperature: 0 °C.

Figure S1 shows that BD absorbs strongly at 240 nm, with some less-intense absorption peaks observed at around 280 and 350 nm. The corresponding CD spectra show several Cotton effects around these absorption bands (Figure 4a). The two enantiomers show identical but mirror-image CD patterns. In this work, we focused on the relatively large Cotton effect provided by Davydov splitting [22] at around 240 nm that corresponds to the absorption maximum of BD.

Figure 4 shows the effects of *G*-CA and TEA on the CD spectra. Focusing on the value of the CD peak at 250 nm, which is related to the absorption at around 240 nm, the presence of TEA was found to increase the difference in between the R- and S-enantiomers. The *G*-CA system without TEA exhibited no significant change in the CD pattern or intensity, which reveals that TEA promotes interactions between *G*-CA and BD, which results in high enantioselectivity.

**Figure 5:**
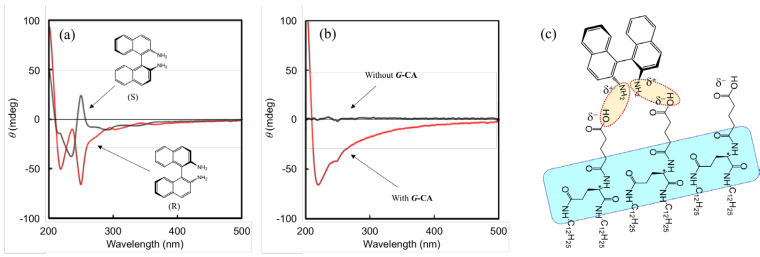
(**a**) CD and (**b**) different CD spectra for *G*-CA systems containing chiral BD. Solvent: 95:5 (v/v) methylcyclohexane:THF. Concentrations: *G*-CA, 0.75 mM; dyes, 0.05 mM; TEA, 0.5 mM. Temperature: 0 °C. (**c**) Illustrating the R-BD-on-*G*-CA (guest-host) interaction. The blue parts show that hydrogen bonding induces secondary chirality with a highly ordered state. The orange parts show multiple interactions of a guest molecule with the highly ordered surface.

Figure 5 summarizes the enantioselectivity results of *G*-CA aggregates for BD. As shown in the difference CD spectra (Figure 5b), the large CD intensity near 200 nm is remarkably enhanced by the addition of *G*-CA. This Cotton effect is most likely due to the three amide bonds around the L-glutamic acid moiety; however, such a large value is generally unusual in a single molecular base; hence, we conclude that it is due to secondary chirality based on highly ordered carbonyl groups that are aggregated through intermolecular hydrogen bonding. On the other hand, the negative value at around 250 nm is due to BD guest molecules with *G*-CA aggregates, and shows that the R-enantiomer interacts more strongly with the *G*-CA aggregates than the S-enantiomer. In addition, fluorescence spectroscopy did not show any significant changes in emission maxima; however, intensities changed slightly, as shown in Figure S2. These results suggest that BD-on-*G*-CA (guest-host) interactions occur near the surfaces of the polar moieties of the *G*-CA aggregates, rather than inside the aggregates; stronger binding to R-BD over S-BD enhances the chirality of both the host aggregate and the guest molecule.

It is difficult to determine why *G*-CA interacts more strongly with the R-enantiomer, but a multiple-interaction mechanism involving *G*-CA and BD should be considered. To support this hypothesis, we did not observe significant selectivity for naphtylmethylamine (Naph), as a single-amine guest molecule, by CD. In addition, selectivity was not observed under conditions in which *G*-CA does not aggregate, for example, at low concentrations below the critical aggregation concentration [10] and at high temperatures (e.g., 60 °C). These observations suggest that enantioselectivity is effectively induced when *G*-CA forms a highly ordered and negatively charged surface in which carboxyl groups are assembled, which provides multiple points of electrostatic interaction for the two amino groups of the guest molecule (Figure 5c).

## 4. Conclusions

In this study, we established that enantioselectivity can be induced by simple COOH functional groups. For this purpose, an L-glutamide-based organogel system was used as the chiral host system, in which COOH groups can integrate to form nanofibrillar aggregates. The resultant highly ordered chiral surface provides multiple interaction sites for guest molecules; as a result, effective enantioselectivity was observed for a diamine but not for a compound containing a single amino group. Its compatibility with polymer materials as well as its compositing ability are advantages of the organogel system [23]. Therefore, by exploiting the formation of enantioselective thin films that take advantage of the features of the organogel system, we anticipate that such organogels will be used in future sensing systems.

## Data Availability

The datasets generated during and/or analyzed during this study are not publicly available but are available from the corresponding author on reasonable request.

## Supplementary Materials

The following are available online at https://www.mdpi.com/article/10.3390/nano11061376/s1. Figure S1: UV-visible spectrum of BD. Figure S2: Fluorescence spectra of BD in the presence of *G*-CA.
