# Supplementary material for: Enantioselective Self-Assembled Nanofibrillar Network with Glutamide-Based Organogelator
Source: Nanomaterials (Basel). 2021 May 23;11(6):1376. doi: 10.3390/nano11061376 (PMC8224585; doi:10.3390/nano11061376)
Supplement: Supplementary file 1 [file nanomaterials-11-01376-s001.zip › nanomaterials-1221696-supplementary.pdf]

Supplementary Materials

Enantioselective Self-assembled Nanofibrillar Network with Glutamide-based Organogelator

Nao Nagatomo[1], Hisashi Oishi[1], Yutaka Kuwahara[1], Makoto Takafuji[1,*], Reiko Oda[2], Taisuke Hamada[3], and Hirotaka Ihara[1,3,*]

[1] Department of Applied Chemistry and Biochemistry, Kumamoto University, 2-39-1 Kurokami, Chuo-ku, Kumamoto 860-8555, Japan
[2] Institut de Chimie & Biologie des Membranes & des Nano-objects, CNRS, Pessac 33607, France
[3] National Institute of Technology, Okinawa College, 905 Henoko, Nago, Okinawa 905-2192, Japan
* Correspondence: ihara@kumamoto-u.ac.jp; Tel.: +81-96-342-3662

Supplementary figures

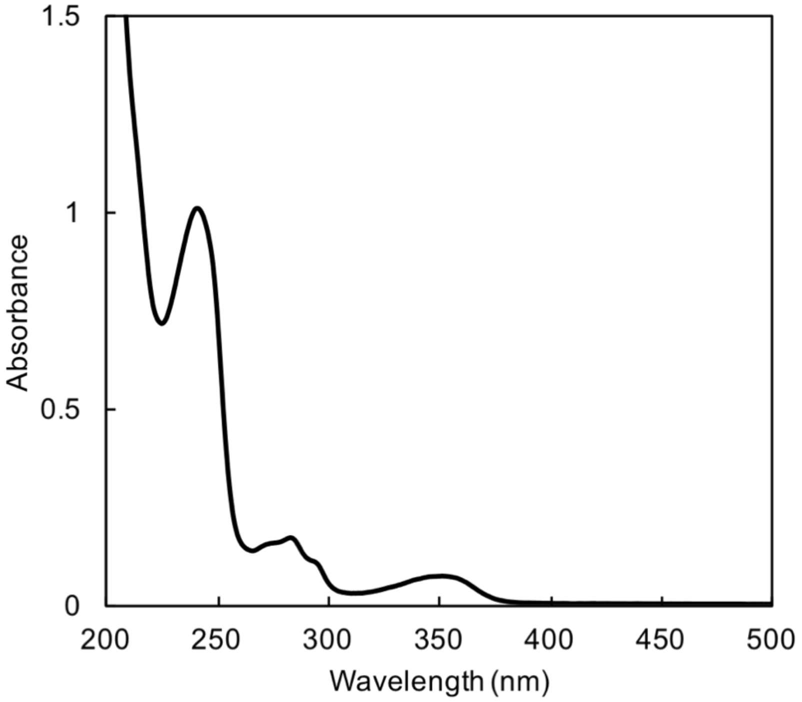

Figure S1. UV-visible spectrum *of* R-BD in 95:5 (v/v) methylcyclohexane:THF. Concentration: 0.05 mM. Temperature: 0 °C.

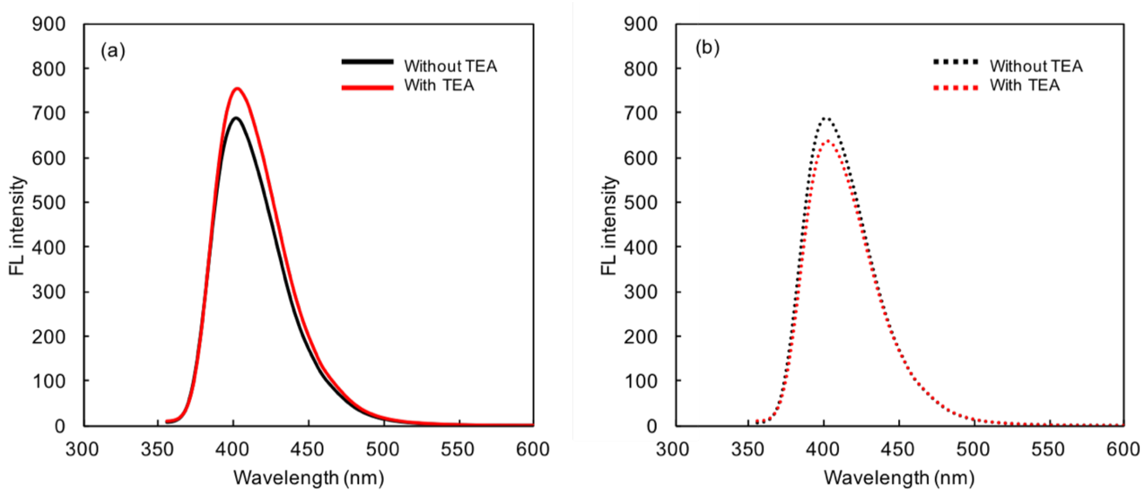

Figure S2. Fluorescence spectra of (a) R-BD and (b) S-BD in 95:5 (v/v) methylcyclohexane:THF containing *G*-CA. Concentrations: *G*-CA, 0.75 mM; BD, 0.05 mM; TEA, 0.5 mM. Temperature: 0 °C. Excitation: 350 nm.
